# Is the Quantity of Capsaicin in Food Related to Its Organoleptic and Sensory Effects? A Systematic Review

**DOI:** 10.1002/fsn3.71407

**Published:** 2026-01-26

**Authors:** Sean Hayward, David J. Leaver, Andrea Crampton

**Affiliations:** ^1^ School of Dentistry and Medical Sciences Charles Sturt University Albury New South Wales Australia; ^2^ School of Dentistry and Medical Sciences Charles Sturt University Wagga Wagga New South Wales Australia

**Keywords:** capsaicin, organoleptic, pain, quantification, scoville heat unit

## Abstract

Capsaicin, an alkaloid predominantly found in plants in the genus *Capsicum*, is naturally present in food and utilized in dietary supplements and medicinal products. It interacts with cellular receptors, triggering a sensory response often perceived as pain, measurable by the Scoville Organoleptic Test. However, due to its susceptibility to biases, this test has largely been supplanted by quantitative methods for determining capsaicin content. This systematic review investigates the relationship between quantitatively measured capsaicin levels in dietary products and their sensory effects. The review protocol, registered with the Open Science Framework (OSF), involved searches in the EBSCOHost, ProQuest, and Ovid databases. Findings indicate a direct correlation between quantitatively determined capsaicin levels and extrapolated Scoville Heat Unit (SHU) values. Additionally, associations were noted between capsaicin exposure and physiological responses, as well as between capsaicin sensitivity and other chemesthetic and taste modalities. However, no direct relationship was found between quantitative capsaicin levels in dietary products and consistent, reproducible measurements of their sensory effects. This research marks a point in the discourse where quantification technology refines the traditional SHU system and underscores the need to advance quantitative detection beyond SHU.

## Graphical abstract

This review examined the link between capsaicin levels and extrapolated Scoville Heat Units (SHU), noting inconsistent sensory correlations. Although precise methods like HPLC quantify capsaicin accurately, converting to SHU reduces reliability. Emerging technologies such as electronic tongues and AI may address these limitations, offering standardized chemesthetic profiling and potentially replacing the SHU scale.

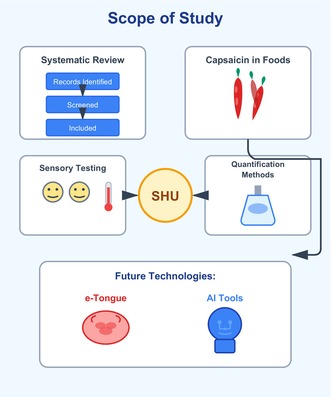

## 1 Introduction

Capsaicin (8‐methyl‐*N*‐vanillyl‐6‐nonenamide) is a pungent vanilloid and alkaloid produced exclusively by plants in the genus Capsicum. Among the 12 identified capsaicinoids in chili peppers, capsaicin is the most abundant, constituting over 60% of the measured capsaicinoid content (Kozukue et al. 2005). Topical application of capsaicin has demonstrated therapeutic benefits in various neuropathic disorders (Bae et al. 2012; Goci et al. 2021; Wolf et al. 1999), including neck pain (Mathias et al. 1995), diabetic neuropathy (Hayman and Kam 2008), and pruritus (Ellis et al. 1993; Hayman and Kam 2008). Noticeably, QUTENZA (capsaicin 8% patch) is approved in the European Union and the United States of America to treat patients with neuropathic pain (Anand and Bley 2011).

Capsaicin fulfills “Food is Medicine” criteria (Adelman and Haushofer 2018; Mozaffarian et al. 2024), however, its medical use needs to be carefully monitored by health care professionals and dietary capsaicin has shown potential in anti‐nausea and anti‐emetic applications (Darmani et al. 2014; Hayman and Kam 2008), as well as benefits against a variety of metabolic disorders (Hochkogler et al. 2018; McCarty et al. 2015), particularly in individuals with a high body mass index (Zsiborás et al. 2018). Other benefits include gastroprotective (Hayman and Kam 2008), cardioprotective (Czikora et al. 2012; McCarty et al. 2015), and neuroprotective applications that may reduce the risk of stroke (Abdel‐Salam and Mózsik 2023; McCarty et al. 2015; Veldhuis et al. 2003). However, overexposure to capsaicin can cause an unpleasant burning sensation, which can be alleviated by consuming dairy foods, fatty foods, bread, or alcohol (Farah et al. 2023).

The traditional method for quantifying capsaicin is the Scoville Organoleptic Test (Scoville 1912), which relies on subjective human evaluations of organoleptic experiences to generate a score known as Scoville Heat Unit (SHU). Despite its continued use, this method has been criticized for its reliance on human tasting panels, lack of statistical rigor and food matrix effect where ratings vary with food combinations (Zhang 2014). Capsaicinoids are known to induce a range of pungency perceptions (Krajewska and Powers 1988). however, the noted variations in the perception of pungency from different *Capsicum* species and varieties tend to indicate that pungency perception is more complicated than just intensity and capsaicin concentration (Borcherding et al. 2025; Guzmán and Bosland 2017; Kostyra et al. 2010). Methods for capsaicin quantification include high‐performance liquid chromatography (HPLC), mass spectrometry, electrochemical analysis, and ultraviolet spectroscopy. This systematic review aims to investigate the association between quantitatively measured capsaicin levels in dietary products and their corresponding sensory/organoleptic effects in addition to highlighting shortcomings of the SHU scale.

## 2 Methodology

The research protocol was registered as a Generalized Systematic Review with OSF (den Van Akker et al. 2024).

### 2.1 Search Strategy

#### 2.1.1 Review Framework

The search strategy utilized a SPIDER framework (Sample, Phenomenon of Interest, Design, Evaluation, Research Type) as outlined by Cooke et al. (2012) to focus the search and guide the development of search criteria (Cooke et al. 2012). The SPIDER framework is recommended over the more common PICO framework for reviews that include both qualitative and mixed‐method research (Cooke et al. 2012) and has been previously used in food technology reviews (Alhujaili et al. 2023; Carducci et al. 2025).

The SPIDER framework addresses each key interrogative concept relevant to the goals of the review. Firstly, it identifies the sample: capsaicin and related capsaicinoids, with specific reference to measuring capsaicin levels in food products. Secondly, it explores the key phenomena of interest: the organoleptic (sensory) and pain‐mediating effects of capsaicin. Additionally, it outlines the conditions under which these phenomena should occur, specifically within dietary products that are introduced into the gastrointestinal tract. Lastly, the framework examines the relationship between measuring capsaicin levels and the associated phenomena. Table 1 shows how the framework was applied to the search for literature that aligned with the research question.

**TABLE 1:** SPIDER based inclusion and exclusion criteria.

Framework	Inclusion	Exclusion
Sample	Capsaicin‐containing foods Capsaicin extract Capsaicin‐based therapeutic products	Non‐capsaicinoids Non‐specific samples
Phenomenon of Interest	Quantity Ratio Binding affinity	Culinary appeal/evaluation Food safety studies
Design	In vivo and In vitro HPLC Detailed preparation	Non‐reproducible methodologies Unknown preparations Secondary data, reviews or meta‐analysis
Evaluation	Comparison between organoleptic indicators and quantitative measures	No organoleptic measures ONLY organoleptic measures
Research type	Since 2013 English language Peer reviewed Quantitative or Mixed studies Case studies Primary data	Exclusively qualitative No quantitative methodology Gray literature Media articles Secondary date, reviews or meta‐analysis

#### 2.1.2 Data Sources

In March 2024, searches were conducted across three key database platforms, EBSCOhost, OVID, and ProQuest, encompassing nine databases. The databases searched in each platform were EBSCOHost‐Academic Search Complete, Food Science Source, Health *Source:* Nursing/Academic Edition; Ovid‐Medline; ProQuest‐Biological Science Database, Health & Medical Collection, Nursing & Allied Health Database, Science Database and Natural Science Collection.

#### 2.1.3 Search Syntax

Each database platform required a unique approach to constructing complex searches. Component search strings were tailored to the syntax of each platform to maintain consistent search focus aligned with the research question. The search syntax for each database is detailed in Table 2. Controlled vocabulary terms used included: EBSCOHost‐ capsaicin, pungency; ProQuest‐ capsaicin, pungency, organoleptic, organoleptic properties, organoleptic effects; Ovid‐ capsaicin, sensation.

**TABLE 2:** Search syntax used for each platform.

Platform	Search syntax
EBSCOHost	SU capsaicin OR TI capsaicin OR AB capsaicin AND TI pungen* OR AB pungen* OR SU pungency OR TI organoleptic OR AB Organoleptic AND pain
ProQuest	SU capsaicin OR TI capsaicin OR AB capsaicin AND SU pungency OR TI pungency OR AB pungency OR SU organoleptic properties OR SU organoleptic evaluation OR SU organoleptic OR TI organoleptic OR AB organoleptic AND pain
Ovid	1 = SU capsaicin 2 = AB TI capsaicin* 3 = 1 OR 2 4 = TW pungent 5 = SU sensation 6 = TW organoleptic 7 = 4 OR 5 OR 6 8 = 3 AND 7 9 = TW pain 10 = 8 AND 9 11 = Limit 10 to year=“2013–2024” 12 = limit 11 to human

Abbreviations: AB, abstract; SU, subject; TI, title; TW, text.

### 2.2 Article Screening

#### 2.2.1 Record Management

Each search result was assigned a unique identifier, prefixed by the first letter of the database platform. Duplicates were removed before the screening. The search parameters, results, and outcomes of title and abstract screenings, as well as full‐text screenings, were documented using “PRIMARY Excel Workbook for a Two‐Person Systematic Review” (VonVille 2015).

#### 2.2.2 Screening Stages

The screening process was guided by the SPIDER framework and conducted in two phases by two independent authors. In phase one, titles and abstracts were reviewed, applying the following exclusion criteria: non‐human subject, non‐quantitative data, non‐dietary focus, and studies without significant emphasis on pain, organoleptic factors, or capsaicin.

In phase two, the full texts of remaining articles were reviewed, applying the same exclusion criteria as phase one but with the additional requirement that the full article be in English. This removed articles from bilingual journals with English abstracts but non‐English full texts.

### 2.3 Thematic Synthesis

A thematic synthesis based on methodology described by Thomas and Harden (2008) was then conducted to facilitate the comparison of information from diverse disciplines (biochemistry, food science, health). After the screening process, the lead author thoroughly read the remaining articles multiple times to identify five descriptive themes that transcended discipline‐specific boundaries. The five themes were: (1) organoleptic descriptors, (2) organoleptic measurements, (3) quantitative descriptors related to capsaicin, (4) quantitative measures of capsaicin content, (5) general data on capsaicin and human sensory aspects. Once the papers were organized into the descriptive theme clusters, further reading identified thematic themes that positioned the research in the context of the descriptive themes and the broader research question.

The minimal requirements for inclusion in each of the descriptive themes were as follows:

1. *Organoleptic Descriptors*—The article described the organoleptic effects of capsaicin, cited methodologies, acknowledged shortcomings, described mechanisms, or covered a combination of these topics.
2. *Organoleptic Measurements*—The article included one or more organoleptic tests that relied on a self‐reported human response methodology, or a combination of self‐reported and materially quantifiable methods, and creates a comparison between the multiple methodologies or between a single methodology and a capsaicin quantification method run in parallel with organoleptic testing.
3. *Quantitative Descriptors of Capsaicin*—The article described more than one capsaicin quantification technique in detail.
4. *Quantitative Measurements of Capsaicin*—The article included one or more capsaicin quantification methods, cited prior research justifying the use of such a methodology, or justified the use of only one quantification methodology. Alternatively, the research had used one or more predetermined capsaicin standards for the purpose of organoleptic testing and provided a detailed justification for the selection of specific concentrations or formulations of capsaicin.
5. *General Information on Capsaicin*—The article described both capsaicin and related receptors in enough detail to provide a rudimentary background understanding to a reader unfamiliar with the subject.

## 3 Results

### 3.1 Screening Results

**FIGURE 1:**
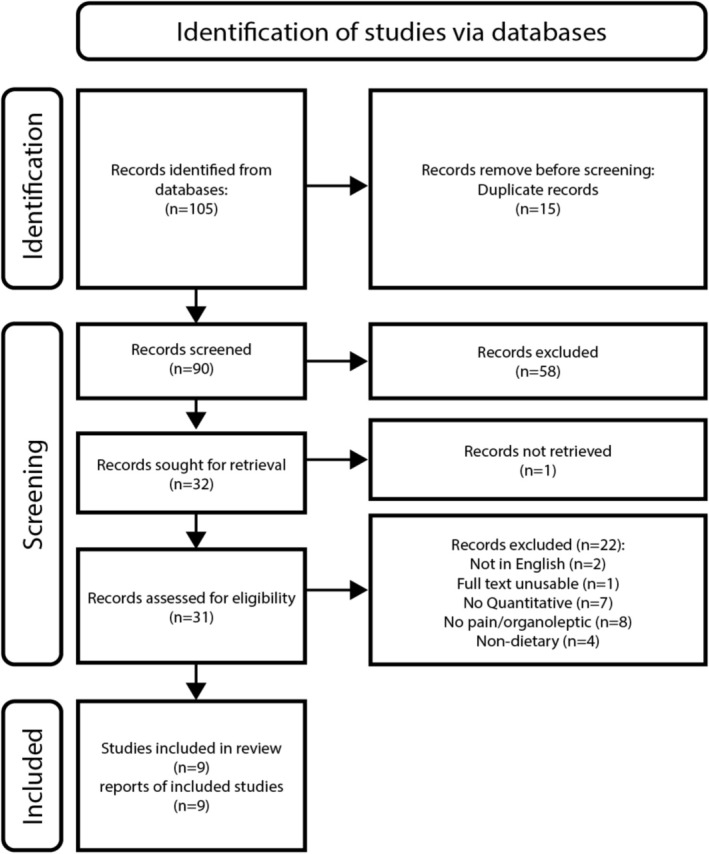
PRISMA flow diagram of screening outcomes.

An initial search across the three platforms yielded 105 articles. After the screening process, this number was reduced to nine articles as illustrated in Figure 1.

During the title and abstract screening stage, Cohen's Kappa was employed to assess inter‐rater reliability, which indicates the level of agreement between screeners. According to established scales, scores above 0.90 signify nearly perfect agreement (Hanegraaf et al. 2024). In our study, Cohen's Kappa for inter‐rater reliability between the two screeners was 0.98, with a confidence interval of 0.93. There was one article where the screeners had differing evaluations; however, after discussion, it was included in the next stage of screening. The final nine articles and the descriptive themes that were evident in them are presented in Table 3.

**TABLE 3:** Included papers and descriptive themes present in each.

Article	Organoleptic descriptors	Organoleptic measurements	Quantitative descriptors	Quantitative measurements	General information
Cunha et al. 2022	✓	—	✓	✓	✓
Beckers et al. 2022	✓	✓	—	✓	✓
Patcharatrakul and Gonlachanvit 2016	✓	✓	—	—	✓
Tsuchihashi et al. 2020	✓	✓	—	✓	✓
Matsuyama et al. 2021	✓	✓	✓	✓	✓
He et al. 2023	✓	✓	✓	—	✓
Kaiser et al. 2015	✓	✓	—	✓	✓
Roukka et al. 2023	✓	✓	—	✓	✓
Roukka et al. 2021	✓	✓	—	✓	✓

### 3.2 Thematic Synthesis Results

At least four of the five descriptive themes were evident in all articles, except Patcharatrakul and Gonlachanvit (2016), which didn't meet the criteria for the quantitative‐centred themes. Further, all themes were evident in the work of Matsuyama et al. (2021).

#### 3.2.1 Organoleptic Descriptors

This theme was present in all articles, with closer analysis identifying nine analytical themes: seven relating to problems with existing testing protocols and two noting possible reasons of solutions. Three articles described multiple problems inherent in organoleptic testing for capsaicin content or pungency (Roukka et al. 2023, 2021; Tsuchihashi et al. 2020). Four articles specifically cited problems inherent in human sensory evaluation with self‐reporting methodologies (Beckers et al. 2022; Cunha et al. 2022; He et al. 2023; Roukka et al. 2023), with two articles citing problems with the original Scoville Organoleptic Test or its derivations by name (He et al. 2023; Matsuyama et al. 2021). Three articles described the physiological mechanisms underlying these problems (Matsuyama et al. 2021; Patcharatrakul and Gonlachanvit 2016; Roukka et al. 2021). Seven articles described the problem of human subjectivity (Beckers et al. 2022; He et al. 2023; Kaiser et al. 2015; Matsuyama et al. 2021; Patcharatrakul and Gonlachanvit 2016; Roukka et al. 2023, 2021), five articles described the problem of desensitization to noxious stimuli after exposure (He et al. 2023; Matsuyama et al. 2021; Patcharatrakul and Gonlachanvit 2016; Roukka et al. 2023, 2021), and three articles described differences in sample preparation (Kaiser et al. 2015; Matsuyama et al. 2021; Roukka et al. 2021). Three articles suggested strategies or developed detailed protocols to address such problems experimentally (He et al. 2023; Matsuyama et al. 2021; Roukka et al. 2023).

#### 3.2.2 Organoleptic Measurements

This theme was present in eight articles with closer analysis identifying four analytical themes relating to the number of methods described, the mechanisms of quantification and emerging research. Seven articles described multiple methods for measuring organoleptic effects (Beckers et al. 2022; He et al. 2023; Kaiser et al. 2015; Matsuyama et al. 2021; Patcharatrakul and Gonlachanvit 2016; Roukka et al. 2023, 2021). Four articles utilized a self‐reported methodology, where human subjects rated, scored, or otherwise evaluated sensory effects after administering capsaicin, similar to the original Scoville Organoleptic Test (He et al. 2023; Roukka et al. 2023, 2021; Tsuchihashi et al. 2020). Meanwhile, four articles employed methods to measure an organoleptic pathway that could yield materially quantifiable results, whether in vivo or in vitro (Beckers et al. 2022; Kaiser et al. 2015; Matsuyama et al. 2021; Patcharatrakul and Gonlachanvit 2016). A phenomenon was deemed “materially quantifiable” if its presence, concentration, intensity, or any combination of these could be measured using instruments independent of human judgment.

Among the articles that reported materially quantifiable measurements, one focused on capsaicin interactions with a receptor, TRPV1 (Matsuyama et al. 2021), while three examined methods based on downstream neurological stimulation (Beckers et al. 2022; Matsuyama et al. 2021; Patcharatrakul and Gonlachanvit 2016). Additionally, four articles utilized both self‐reported and materially quantifiable organoleptic measurements (Beckers et al. 2022; Kaiser et al. 2015; Matsuyama et al. 2021; Patcharatrakul and Gonlachanvit 2016).

Of the articles that performed more than one measurement of organoleptic effect, two demonstrated a logical relationship between the tests conducted (Beckers et al. 2022; Matsuyama et al. 2021). Furthermore, four articles introduced a novel approach to organoleptic testing (Beckers et al. 2022; Matsuyama et al. 2021; Roukka et al. 2023, 2021).

#### 3.2.3 Quantitative Descriptors of Capsaicin

While all articles underwent some aspect of quantitative analysis as part of the initial screening process, only three articles were examined in depth for this descriptive theme. Although there were only three articles, this theme was still considered important to highlight and examine further, as it was a central aspect of the research question and considered more amenable to comparison across levels of discipline‐specific technical application. No formal analytical themes were developed; however, interesting clusters were apparent. Three articles presented more than one method for determining capsaicin content in food (Cunha et al. 2022; He et al. 2023; Matsuyama et al. 2021). Additionally, one article examined the benefits and drawbacks of several techniques (Matsuyama et al. 2021).

Two articles emphasized the importance of separating capsaicinoids from food products during experimental procedures (Cunha et al. 2022; Matsuyama et al. 2021). One article described how interfering compounds can affect the processing of dietary products before capsaicin determination. Another article raised concerns about the reliability of methods when capsaicinoids are extracted prior to analysis, suggesting that this could misrepresent the original pungency of the dietary product (Matsuyama et al. 2021).

Furthermore, two articles proposed strategies to address methodological shortcomings in current quantification techniques (Cunha et al. 2022; Matsuyama et al. 2021), while one article suggested or demonstrated novel approaches to capsaicin quantification (Beckers et al. 2022). An approach was classified as “novel” if it had not been encountered during the review process or if the authors explicitly referred to it as a novel technique.

#### 3.2.4 Quantitative Measurements of Capsaicin

Seven articles included aspects that aligned to this descriptive theme. These were further grouped relative to the use of direct measurements or application of a standard. Two articles utilized specific techniques for determining capsaicin content (Cunha et al. 2022; Kaiser et al. 2015). The other seven articles did not provide a method for quantifying capsaicin, justifying this absence by relying on predetermined capsaicin standards for their organoleptic testing.

#### 3.2.5 General Information on Capsaicin

This general descriptor captured secondary elements of the topic that were considered important to consider, yet not central to the question's theme of quantification. Four described the synthesis of capsaicin (Cunha et al. 2022; He et al. 2023; Kaiser et al. 2015; Roukka et al. 2021), three described the chemical structure of capsaicin (Cunha et al. 2022; He et al. 2023; Kaiser et al. 2015), and four described the biological (organoleptic or chemesthetic) activities of capsaicin (Cunha et al. 2022; He et al. 2023; Kaiser et al. 2015; Patcharatrakul and Gonlachanvit 2016).

## 4 Discussion

The discrete focus of our investigation and the time periods covered by the selection criteria (2013–2024) resulted in a limited number of relevant papers for our analysis. Although several seminal works may have been excluded due to systematic review methodology, a broad range of articles and seminal papers were reviewed during the development of the research question and the contextualisation of the findings.

The review of how the different components of the research question as evidenced among the descriptive themes and the sub groupings within revealed an apparent imbalance in how the literature addressed key concepts related to the primary research question. The articles with more of a focus on the organoleptic aspects rarely examined the quantitative aspects in depth, and vice versa. This discrepancy suggests that while the core concepts encapsulated in our research question were well represented across the literature reviewed, their combination was limited. This has implications for the food technology industry, as the lack of reliable research‐backed connections between levels of capsaicin and consumer experience may impede innovative use of capsaicin substances despite advancements in technology for assaying, compiling and delivery.

Notably, only two articles (Cunha et al. 2022; Kaiser et al. 2015) included a capsaicin quantification method, while seven included organoleptic measurements. Cunha et al. (2022) used HPLC coupled with a UV–VIS detector to determine levels of capsaicinoids in food products available in Brazilian supermarkets. The HPLC output was converted to SHU based on a conversion factor of 16 after Todd et al. (1977). Kaiser et al. (2015) directly related quantitative measures (via HPLC‐UV/VIS) of capsaicin with organoleptic experiences as measured by subjective reports of pungency from a trained tasting panel. While the focus of their research was on nanoencapsulation of capsaicin to enhance its utility in food and medicinal preparations, the combination of a tasting panel and quantitative measures echoed the original work on relating quantitative measures to pungency values, namely SHU (Todd et al. 1977). Todd et al. (1977) determined the process for converting quantitative measures of capsaicin into the more familiar SHU units (ppm X 16.1) used widely in the industry, including by Cunha et al. (2022). However, Cunha et al. (2022) noted that other authors have used other conversion factors ranging from 15 to 16.1, highlighting ongoing inconsistency in converting highly sensitive quantitative measures into the more subjective but better‐known SHU system.

Interestingly, Todd et al. (1977), noted a gender difference among their panel of trained tasters, further highlighting the truly subjective nature of the SHU scale. These inconsistencies in the context of our research question highlight an important gap in the literature as it seems the food technology industry lacks a reliable means to communicate potential organoleptic effects of capsaicin formulations to a diverse user base. Current reliance on conversion to SHU may dilute the accuracy of information from precise instrumentation and result in formulation decisions that may fail in some markets.

Current protocols for measuring capsaicin from purified samples offer significant advantages over traditional SHU and early gas‐chromotography analyses, such as cost‐effectiveness, improved sensitivity, and portability. However, several compounds can influence the perception of pungency in food products (He et al. 2023). Understanding the role of interfering compounds in the perception of pungency may require a broader analysis of pungent foods. This is where the e‐tongue technology and applications may provide the missing link as they can identify levels of capsaicin and predict the human sensory experience without the subjective nature of tasting panel and tasting fatigue, while still allowing translation into SHU (Ahmed et al. 2024; Roukka et al. 2023; Schlossareck and Ross 2019).

Most reported articles focused on exploring the organoleptic effects of capsaicin, either alone or alongside other common dietary compounds. Understanding how capsaicin influences the overall eating experience is crucial for developing strategies to promote its adoption for inherent health benefits and therapeutic potential. The methods used for organoleptic testing varied significantly in scope and technique. Most articles discussing the physiological effects of capsaicin primarily evaluated its organoleptic effects. This included methods to quantify pungency and assessments of how various chemesthetic modalities impact the perception of taste and vice versa. Two articles specifically concentrated on measuring organoleptic effects in clinically significant populations (Patcharatrakul and Gonlachanvit 2016; Tsuchihashi et al. 2020).

Other articles investigated potential relationships between sensitivity to various chemesthetic and taste modalities. Each study suggested that sensitivity in one modality might indicate sensitivity in another (He et al. 2023; Roukka et al. 2023, 2021). Foods high in capsaicin were shown to enhance the perception of various taste modalities, enriching the flavor of pungent foods. A reciprocal phenomenon was observed, where different taste modalities influenced the perception of capsaicin‐induced pungency.

Two articles explored methods to quantify the organoleptic properties of capsaicin by measuring measurable phenomena associated with capsaicin activity (Beckers et al. 2022; Matsuyama et al. 2021). The pathways through which capsaicin induces a pain response are complex and open several avenues for both qualitative and quantitative investigations (Caterina et al. 1997; Thouaye and Yalcin 2023; Zhang et al. 2023). Treating neuropathic pain is incredibly challenging, and prescribed treatments are inherently complex too (Peppin and Pappagallo 2014). Capsaicin exhibits minimal side effects based on its topical administration, and a local anesthetic is applied prior to the administration of 8% capsaicin patches to ameliorate intense burning sensations (Thouaye and Yalcin 2023).

One article examined the relationship between capsaicin‐induced pain responses and subsequent neurological stimulation using a visual analogue scale (VAS) alongside functional magnetic resonance imaging (fMRI) (Beckers et al. 2022). Participants were given a duodenal infusion containing capsaicin or a saline control and asked to rate their pain responses during the infusion process. The reported VAS responses were compared to contemporaneous fMRI images to analyze potential relationships between capsaicin and neurological signaling.

The evaluation of capsaicin quantification protocols and organoleptic testing methods outlined in relevant articles suggests possible future directions for investigating and quantifying the effects of capsaicin on taste and smell. The methods for quantifying capsaicin discussed in this review have proven to be reliable and repeatable when determining the total capsaicinoid profile of food products.

Implementing human sensory evaluation protocols for assessing organoleptic properties introduces a different set of biases. The Scoville Organoleptic Test, used to determine SHU, is a simplified taste test for capsaicin. Due to subjective biases in taste testing, HPLC was adopted for capsaicin determination (He et al. 2023). Additionally, the variability in human sensitivity to capsaicin complicates the use of sensory evaluation. Significant differences in human sensitivity to chemesthetic agents have been documented (He et al. 2023; Roukka et al. 2023; Tsuchihashi et al. 2020), and various mechanisms influence an individual's sensitivity to capsaicin over both short and long terms (He et al. 2023; Patcharatrakul and Gonlachanvit 2016; Roukka et al. 2021). As a result, human sensory evaluations tend to demonstrate poor reproducibility. It remains unclear why SHU measurements that lack consistency and specificity are still used to report on capsaicin chemesthesis. Looking forward, we anticipate the widespread adoption of more advanced technological tools such as the e‐tongue, e‐nose/bionic electronic nose and the incorporation of artificial intelligence/machine learning to examine chemesthesis and sensory profiles for pungent compounds like capsaicin (Chen et al. 2025; Gupta et al. 2025; Martinez‐Velasco et al. 2023; Zhang et al. 2023). More research needs to be done to make these platforms smaller, cheaper and more accessible for consumers and food scientists.

## 5 Conclusion

A systematic review of the literature revealed a connection between experimentally measured capsaicin levels in dietary products and the reported Scoville Heat Unit values.

However, no method to validate these results against quantitative measurements of capsaicin levels in dietary products was utilized. Additionally, sensitivity to capsaicin exposure was found to correlate with sensitivity to other sensory modalities and overall chemesthetic sensitivity. Consequently, the literature did not establish a direct association between quantified capsaicin levels in dietary products and their corresponding sensory effects.

Capsaicin has demonstrated several health benefits; however, the pain response associated with capsaicin consumption can negatively impact its adoption. Nevertheless, QUTENZA is used to treat patients with neuropathic pain and is part of the “Food is Medicine” arsenal. Understanding how foods containing capsaicin induce a pain response and which factors can affect this response could direct future research aimed at developing strategies to promote the therapeutic adoption of capsaicin. We anticipate that combining machine learning/artificial intelligence with e‐tongue/e‐nose technology will 1 day enable chemesthetic analyses for capsaicin to be standardized thus making the SHU scale obsolete.

## Author Contributions

**Sean Hayward:** conceptualization (equal), data curation (lead), formal analysis (lead), investigation (lead), methodology (equal), writing – original draft (equal), writing – review and editing (supporting). **David J. Leaver:** conceptualization (equal), methodology (equal), project administration (equal), supervision (equal), writing – original draft (supporting), writing – review and editing (supporting). **Andrea Crampton:** conceptualization (equal), data curation (supporting), formal analysis (supporting), methodology (equal), project administration (equal), supervision (equal), validation (equal), writing – original draft (supporting), writing – review and editing (lead).

## Disclosure

The authors have nothing to report.

## Data Availability

Data sharing is not applicable to this article as no datasets were generated or analyzed during the current study. All articles selected for the review are noted in the reference list and available via academic databases.
